# Laser-fabricated cell patterning stencil for single cell analysis

**DOI:** 10.1186/s12896-017-0408-8

**Published:** 2017-12-19

**Authors:** Jacob J. Messner, Honor L. Glenn, Deirdre R. Meldrum

**Affiliations:** 1 OSIsoft, 1600 Alvarado St, San Leandro, CA 94577 USA; 2 Biodesign Center for Immunotherapy, Vaccines, and Virotherapy, The Biodesign Institute, Arizona State University, 1001 S. McAllister Ave, Tempe, AZ 85287 USA; 3 Center for Biosignatures Discovery Automation, The Biodesign Institute, Arizona State University, 1001 S. McAllister Ave., P.O. Box 877101, Tempe, AZ 85287-7101 USA

**Keywords:** Single-cell, Microwell, Cell patterning, Adhesion, Cell culture

## Abstract

Precise spatial positioning and isolation of mammalian cells is a critical component of many single cell experimental methods and biological engineering applications. Although a variety of cell patterning methods have been demonstrated, many of these methods subject cells to high stress environments, discriminate against certain phenotypes, or are a challenge to implement. Here, we demonstrate a rapid, simple, indiscriminate, and minimally perturbing cell patterning method using a laser fabricated polymer stencil. The stencil fabrication process requires no stencil-substrate alignment, and is readily adaptable to various substrate geometries and experiments.

## Background

The ability to manipulate and selectively localize cells into patterns or distinct microenvironments is critical for single cell analysis [1–4], tissue engineering [5, 6], cell signaling studies [7–9], drug screening [10–12], and cell migration assays [13, 14]. Exploring the population dynamics and communal contributions within heterogeneous cell populations is fundamental to furthering our understanding of disease pathology [15–18]. In recent years, much effort has been focused on developing innovative, active and passive cell patterning methods and applications thereof. Many active cell patterning and isolation methods utilize microfluidic systems, in which cells are manipulated and transported using fluidic forces. Inkjet-based cell ‘printing’ and deposition methods have proven effective at sorting and patterning cells at the bulk and single cell level, but are typically low throughput and raise concerns about cell stress responses [19–22]. A variety of microfluidic geometries have been used to pattern cells into hydrodynamic traps at single cell capture efficiencies nearing 100% for capture rates on the order of thousands of cells per minute [23–29]. While trap-based approaches are very high throughput, they may discriminate against particular cell morphologies or sizes with relevance for human disease [30]. Microfluidic trap environments also impose difficulties in delivering single cells to isolated microenvironments for further experimentation. Droplet based microfluidics, which encapsulate single cells within medium-oil emulsion droplets, are highly effective at isolating cells at hundreds of droplets per second [31–33] and are cost-effective for biomolecular analysis of single cells. However, these approaches are poorly suited for studying temporal processes in live cells due to the limited supply of gas and nutrients in the droplet environment. It is also unclear how droplet technology can be integrated with on-chip analysis that require multistep processes such as single cell PCR [34]. An additional shortcoming of all microfluidic patterning and isolation approaches is that they subject cells to shear stress that can effect cell health, function, and gene expression [35].

Several non-hydrodynamic methods have also proven effective at actively patterning cells. Magnetic spot microarrays can localize magnetically labelled cells onto complementary features of cell patterning substrates [36, 37]. Non-uniform electric fields have been demonstrated to polarize single cells thus creating a mechanism by which they can be patterned or even rotated in the absence of a label [38–40]. Laser and optical fiber based systems have been used to assemble, sort, and pattern live cells [41–43]. A prominent concern with these optical approaches is the large power output required to trap cells and the physiological damage that cells may incur due to heating [44]. Recently, fluidic devices utilizing acoustic fields have proven effective at spatially patterning [45, 46], and rotating [47] cells with 5 × 10^5^ times lower power exposure than optical systems [48]. However, all of these approaches require specialized equipment and expertise at the implementation step.

Many passive cell patterning methods achieve localization through chemical [49–52] or topographical [53, 54] surface modifications, deterring adhesion to undesired regions and/or promoting adhesion to desired regions. This preferential adhesion patterning strategy has also been demonstrated with dynamic substrates, where surface properties can be modulated in real-time to alter adhesion susceptibility [55]. However, substrate surface modification is prone to select for cells with a particular adhesive behavior, and may discriminate against certain phenotypes [56]. A large body of evidence suggests that the distribution of adhesive phenotypes within cell populations has profound implications in biological development and disease pathology [57, 58]. The biased nature of surface modification cell patterning suggests it may be ill suited for high throughput single cell analysis methods where isolation of representative populations is desirable. Further, it is well understood that extra-cellular matrix components that promote cell adhesion also profoundly influence cell physiology [59, 60]. Other passive patterning strategies utilize traditional random seeding approaches but with physical barriers (stencils) to pattern cells onto accessible regions of substrates [61–65]. Because stencil patterning relies upon physical barriers, there is little to no phenotypic discrimination imposed upon the seeded population, so long as the stencil through-holes are large enough to be cell-size indiscriminate. However, the use of a cell patterning stencil to seed cells into predefined features of a substrate typically requires microscale stencil-substrate alignment, presenting a challenge in the utilization of typical stencils.

Therefore, despite significant recent advances, major challenges remain in obtaining large, representative quantities of isolated single cells. Here, we demonstrate the use of a rapidly produced laser-fabricated polymer stencil to pattern cells into wells of microarrays without the need for stencil-substrate alignment. Laser ablation is used to create holes in the polymer film, while it is immobilized and stretched across the microwell array. The stencil remains fixed relative to the underlying substrate during cell seeding. Seeded cells can only access the microwell array by settling through the holes, and are blocked by the film from accessing other areas of the substrate. In our geometry, this results in cells being restricted to the inside of the microwells. After cell adhesion, the stencil is removed and the patterned cells are ready for experimental treatments and analysis. This approach does not require specialized expertise or equipment for the implementation steps. Further, it does not introduce any additional stress to the cells relative to normal cell culture and does not decrease cell viability. We demonstrate that this method greatly increases efficiency of seeding cells into defined locations on a substrate.

## Methods

### Device fabrication

Fused silica wafers (4 in. diameter, 500 μm thickness) were etched using standard photolithography to create 37 arrays of 2980 microwells (20 μm deep, 80 μm inner diameter, 120 μm outer diameter) hexagonally packed within 9 mm by 9 mm footprints. The wafer was partitioned into the 37 individual microwell array cell-seeding substrates using a dicing saw. The substrates were sonicated for 30 min in 1X alkaline detergent to remove particles and fibers, followed by rinsing and sonicating in deionized water for an additional 30 min. Substrates were then dried in a 105 °C oven and stored for later use.

**Fig. 1:**
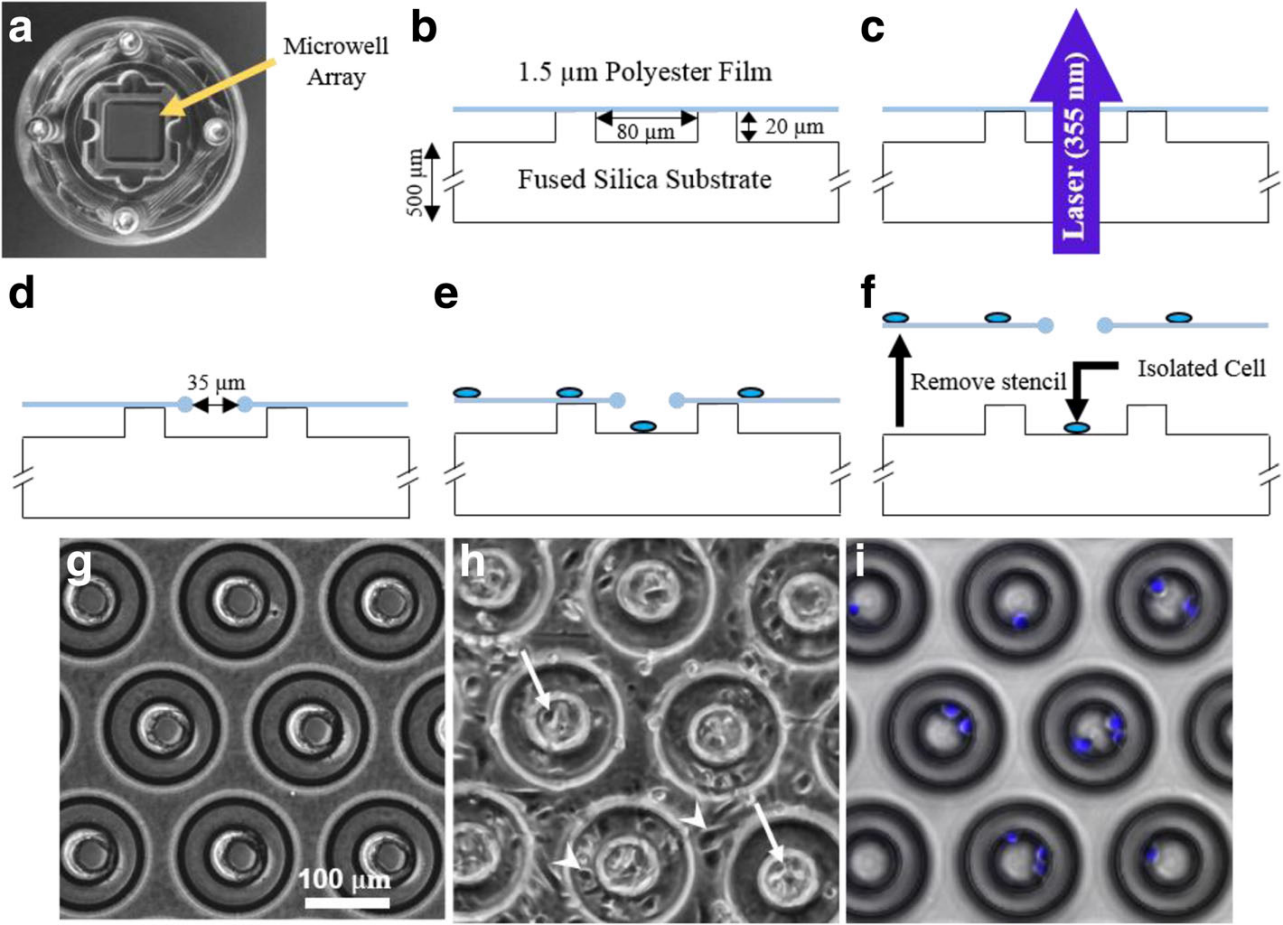
Fabrication process and application of cell patterning stencil. **a** Photograph of the purpose-built fixture (shown inside a 35 mm petri dish) used to secure the polymer thin-film to the microwell array substrate. The microwell array is inside the central reservoir under the polymer film (arrow). **b** Enlarged cross-sectional diagram of a single microwell (80 μm inner diameter) from the microwell array substrate. The polymer thin-film (blue) is secured across the top of the microwells. **c** Laser is focused onto the surface of the thin-film and aligned with the center of each microwell. **d** Thin-film stencil with 35 μm laser fabricated pore aligned with underlying microwell. **e** Traditional random seeding is used; cell suspension is deposited onto the thin-film polymer stencil. Cells (blue ovals) settle onto the stencil surface and through the pores into the microwells below. **f** After cell attachment and spreading, the stencil is removed from the substrate revealing spatially-patterned cells. **g** Phase-contrast image of the stencil substrate prior to cell seeding (equivalent to panel d diagram). **h** Phase-contrast image of the stencil and substrate after cell seeding (equivalent to panel e). Arrows indicate cells inside microwells, arrowheads indicate cells adhered to the stencil. **i** Composite fluorescent image of cell nuclei and phase-contrast image of the microwell array substrate after removal of the stencil (equivalent to panel f)

X-Ray Fluorescence (XRF) film (3090, Chemplex, Palm City, FL) was stretched using an XRF sample cup, rinsed with ethanol, dried with nitrogen, and secured to the surface of the microwell array substrate. A purpose-built fixture was used to clamp the polymer film onto the microwell array substrate and also provide a reservoir for holding cell culture medium during equilibration and cell seeding (Fig. 1a, b). The area of the reservoir footprint that cells are seeded onto was 236 mm^2^. The fixture ensures that the position of the film and underlying substrate are fixed relative to one another through the fabrication and cell seeding process. The fixture was secured to the mechanical stage (ATS250, Aerotech, Pittsburgh, PA) of a laser fabrication environment where a 355 nm UV laser (AVIA 355–3000, Coherent, Santa Clara, California) was focused through the substrate and onto the surface of the polyester XRF film (Fig. 1c). The laser was tuned to output radiation below the ablation threshold of fused silica (48 μJ/pulse, 40 ns pulse width, 6 kHz repetition rate, defocused to 30 μm) to avoid substrate ablation, while still having sufficient energy to form a pore in the XRF film. Poration is achieved through the redistribution of film away from the site of exposure (through ablation or heat induced polymer restructuring) leaving a pore slightly larger than the irradiated area (~35 μm) (Fig. 1d).

The fabrication environment was programmed to traverse the geometry of the cell seeding substrate, aligning to the center of each well and perforating the film through 500 ms UV exposures. The porated substrate assembly was then placed into a 35 mm petri dish and plasma treated to hydroxylate the surface of the substrate and XRF film, sterilize the cell seeding surface, and promote cell adhesion [66].

### Cell culture

Cells were purchased from ATCC (Manassas, VA). MDA-MB-231 (triple negative, metastatic breast cancer, HTB-26) were grown in DMEM supplemented with 10% FBS, 100 units/mL penicillin, and 100 μg/mL streptomycin, HME1 (derived from non-cancerous human breast epithelium, CRL-4010) were grown in MEBM supplemented with 100 units/mL penicillin, 100 μg/mL streptomycin, and supplement and growth factor kit supplied by the manufacturer (CC-3151, CC-4136, Lonza Basel, Switzerland), K562 (chronic myelogenous leukemia, CCL-243) were grown in RPMI supplemented with 10% FBS, 100 units/mL penicillin, and 100 μg/mL streptomycin. All cultures were maintained in a 37 °C, humidified incubator. Adherent cells were collected by trypsinization and all cells were counted and viability assessed with a Countess® Automated Cell Counter (Life Technologies, Carlsbad, CA) using the Trypan Blue dye exclusion assay. Cells were only used if initial viability was >95%.

### Cell seeding

Prior to use, plasma treated fixtures were equilibrated with cell culture medium at 37 °C for 4–18 h. Medium was then removed by pipetting and 600 μL of cell suspension at the indicated concentration was added to the reservoir (Fig. 1e). Cells were allowed to adhere to the substrate for 18 h under normal culture conditions. Then the fixtures were disassembled and the stencil film was peeled off with forceps, effectively removing cells not localized to well-interiors (Fig. 1f). The disassembled fixtures were disinfected with 70% ethanol, rinsed 3× with dH_2_O, then air dried for reuse. The stencil films were discarded after a single use. Substrates were visually inspected prior to cell seeding to verify that the stencil and microwell array were well aligned (Fig. 1g). A second visual inspection was performed prior to removing the stencil film to qualitatively evaluate cell health and morphology on the stencil film, and within the wells (Fig. 1h). A similar procedure was used to assemble the no-stencil control assays wherein the microwell array substrate was placed atop the polymer film inside the fixture. Cells were then seeded into the reservoir onto the uncovered substrate.

### Cell labeling and imaging

Cell viability was evaluated by LIVE/DEAD® Cell Imaging Kit (488/570) (R37601, Thermo Scientific, Waltham, MA) according to the manufacturer’s protocol. Cells were labeled after the 18 h adhesion period and prior to removal of the seeding stencil. In order to evaluate the distribution of cells on the microwell array substrate, cell nuclei were labeled with the NucBlue® Live ReadyProbes® Reagent (R37605Thermo Scientific). The fixtures were disassembled and the stencil film removed as described, then the microwell array substrates were transferred to a six well plate containing fresh cell culture medium and the nuclear label. Cells were incubated for 10 min and then imaged via wide-field microscopy (Fig. 1i). The microwell array was imaged by phase contrast and the nuclear and viability stains were imaged via wide-field epi-fluorescence. The excitation and emission wavelengths in nm were: 360/460 to detect nuclei (blue), 485/540 to detect live cells (green), and 540/600 to detect dead and dying cells (red). All images were collected using an inverted, Nikon TE2000-U fluorescence microscope with a 4× plan apo lens, NA = 0.2, and a Hamamatsu Orca Flash 4.0 digital CMOS camera. The entire microarray was imaged in each channel by stitching individual image fields. Image acquisition was automated using NIS-Elements software.

### Image analysis

Cell viability and localization were evaluated by counting cells from 400 wells from each microwell array. The 400 wells are a randomly sampled subset from the total of ~2980 well detections provided by a normalized 2D cross correlation algorithm implemented in National Instruments LabVIEW software. For cell distribution, labeled nuclei were identified as localized to the interior or the exterior of each well. The exterior of the well was defined as the outside of the well or the lip of the well. Cells located outside of wells were automatically assigned to the nearest well by comparing the Euclidean distances between the cell and the centroids of the 400 sampled wells. The efficacy of the cell patterning stencil was evaluated by calculating the localization efficiency, which was defined as the percentage of cells localized to the interior of wells relative to the total cell count. The biocompatibility of the polymer stencil and the stencil removal process was evaluated by examining the viability of cells in wells, seeded with or without a stencil. Dead (or dying cells) were determined as the percent of red-labeled cells relative to total (blue) nuclei. Live cells were visually confirmed by the presence of green-labeled cytoplasm, however, this label was not used for quantification.

### Statistical analysis

Data were pooled from a minimum of three independent experiments. Data were analyzed by Student’s t-test and Mann-Whitney U test using the R statistical computing environment. *P*-values of <0.05 were considered significant.

## Results

### Biocompatibility of polymer film stencil

**Fig. 2:**
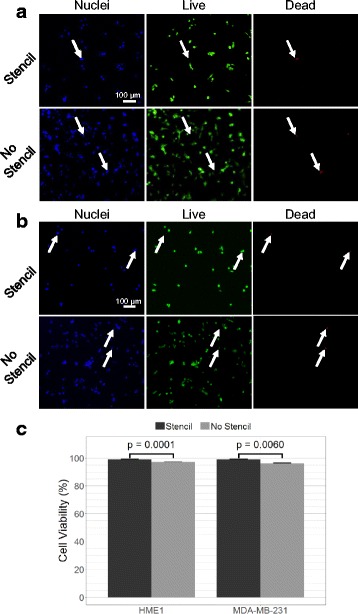
Viability of cells seeded through stencil. **a** Fluorescent micrograph of HME1 cell nuclei (blue), live cells (green), and dead/dying cells (red) seeded onto a microwell array substrate with and without a stencil. Cells were imaged after cell adhesion and removal of the stencil film. Arrows indicate equivalent positions in each color channel. **b** Fluorescent micrograph of MDA-MB-231 cell nuclei (blue), live cells (green), and dead/dying cells (red) seeded onto a microwell array substrate with (top) and without (bottom) a stencil. Arrows indicate equivalent positions in each color channel. **c** Cell viability of HME and MDA-MB-231 cells seeded with and without stencil. Data pooled from 12 independent experiments. Error bars indicate the SEM

We used a commercially available cell viability assay to investigate the biocompatibility of the laser fabricated polymer stencil. We compared viability of cells seeded through the stencil to cells seeded directly onto an uncovered microwell array. Biocompatibility was measured in terms of cell viability, defined as the percentage of cells within wells that were neither dead nor dying, as indicated by absence of red labeling of the nuclei. Cells were manually counted in the blue (all nuclei) and red fluorescent images. The red, dead cell, channel was used for counting since this signal is confined to the nucleus, and thus there is negligible overlap or contact between individual cells, allowing greater accuracy in counting. Figs. 2a and b show representative images of nuclei and LIVE/DEAD labeled HME1 and MDA-MB-231 cells respectively. The stencil reduced the total number cells because it blocks the majority of cells from adhering to the substrate. Use of the stencil did not reduce cell viability in either cell line; in fact, the stencil seeded cells had a very slight increase in survival. For both the stencil and no-stencil seeding environments, cell viabilities were found to be in excess of 95% on average across all experimental seeding densities (Fig. 2c).

### Cell patterning localization efficiency

**Fig. 3:**
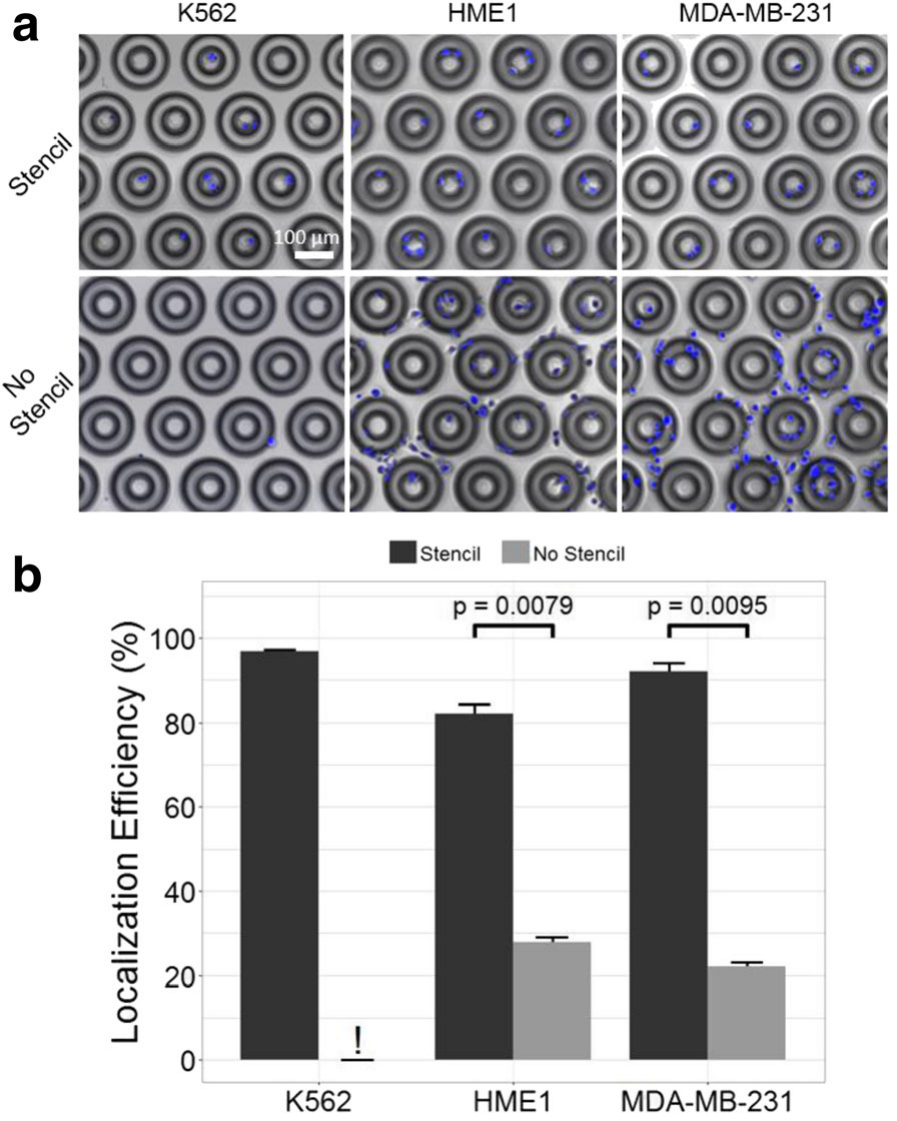
Cell seeding localization efficiency. **a** Composite fluorescent (nuclei, blue) and phase contrast (microwells) micrographs of K562, HME1, and MDA-MB-231 cells seeded with (top) and without (bottom) a polymer stencil. Cells were imaged after cell adhesion and removal of the stencil film. **b** Mean and standard error of localization efficiency for substrates seeded at 100 K cells/mL with and without a polymer stencil. Exclamation point (!) indicates a lack of reliable data due to cells being non-adherent in the absence of the stencil. Data were pooled from four independent experiments. *P*-values determined by Mann-Whitney test

To evaluate the effectiveness of the cell patterning stencil for localizing cells, localization efficiencies were compared between stencil-seeded and directly-seeded substrates. Cells seeded at 100 K cells/mL through a stencil were highly localized to well-interiors (Fig. 3a) with localization efficiencies of 97% for K562 cells, 82% for HME1 cells, and 92% for MDA-MB-231 cells. The stencils increased the percentage of cell in microwells by approximately 3-fold and 4-fold for HME1 and MDA-MB-231 cells, respectively. This represents a significant increase in localization efficiency (*p* < 0.01) relative to controls seeded without stencils (Fig. 3b). Seeding through the stencil had an even more profound effect on the cell distribution of K562 cells. This cell line is generally considered non-adherent and is grown in suspension culture. As expected, when these cells were seeded onto bare microwell array substrates, few cells were observed after the substrate was removed from the fixture and placed in fresh medium in a petri dish. Generally, 0–5 cells were observed across the entire array. Interestingly, when these cells were seeded through the stencil, many cells were retained in the microwells after removal of the stencil and transfer to fresh medium. The mechanism for this is not completely clear, but one possibility is that the stencil reduces turbulence of the medium close to the substrate surface. With this reduction in fluid motion, some very low level of adhesion to the substrate is sufficient to immobilize these cells in the microwells. It should be noted that though they are retained through the disassembly of the fixture and transfer to fresh medium, rinsing these cells with moderate force, or multiple medium changes will dislodge them.

### Effects of seeding density

**Fig. 4:**
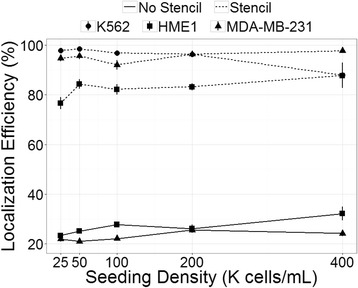
Localization efficiency variation across cell line and seeding density. Localization efficiency was calculated as the percent of cells in microwells relative to total cells on the substrate after removal of the stencil film. Four hundred random wells with surrounding area were sampled from each array. No data were collected for K562 cells in the No Stencil condition, because few or no K562 cells adhered to the substrate without the stencil. Data were pooled from four independent experiments. Error bars indicate SEM

We found that the enhanced localization efficiency provided by the seeding stencil was relatively independent of seeding density with a 4-fold average increase in localization across all seeding densities for MDA-MB-231 cells and 3-fold increase for HME1 cells (t-test; *p* < 0.0001) (Fig. 4). These findings confirm that the stencil is highly effective at controlling the localization of cells using various cell lines and across a wide range of seeding densities.

**Fig. 5:**
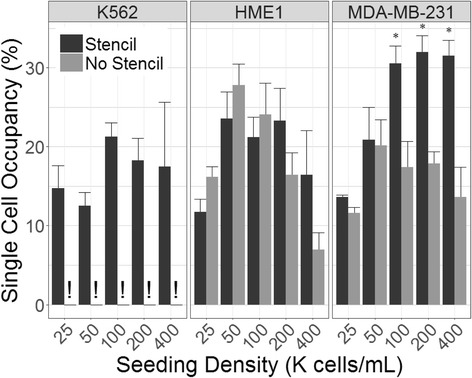
Frequency of single-cell occupancy. Single cell occupancy is presented as the percentage of microwells containing a single cell. Exclamation point (!) indicates a lack of reliable data due to cells being non-adherent in the absence of the stencil. For MDA-MB-231 cells, the stencil significantly increased single-cell occupancy, as indicated by an asterisk, at seeding densities of 100 K cells/mL (*p* = 0.032), 200 K cells/mL (*p* = 0.0095), and 400 K cells/mL (0.019). Four hundred random wells were sampled from each array. Error bars indicate SEM

**Fig. 6:**
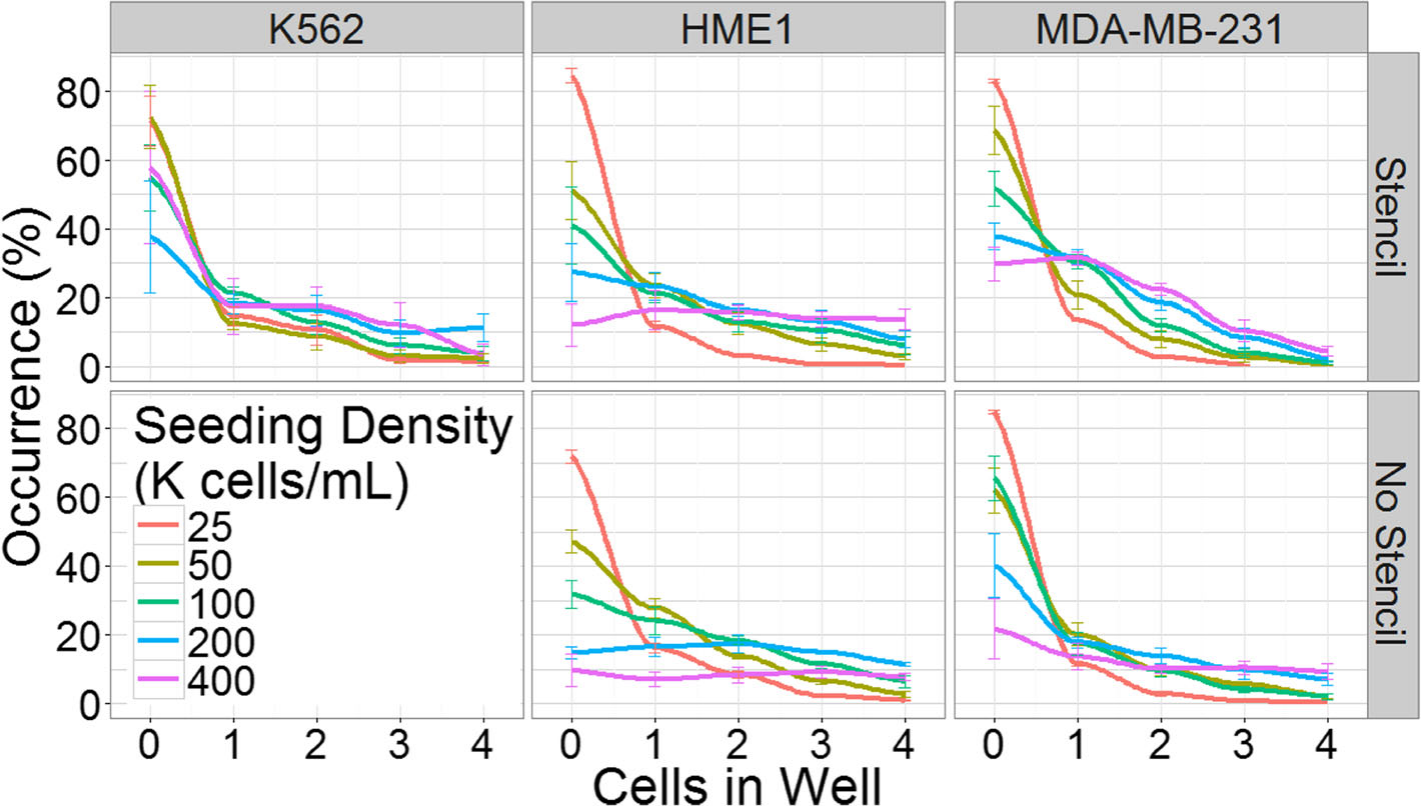
Well occupancy distributions. The occurrence (y-axis) represents the percentage of wells containing a specified number of cells (0–4, x-axis) at each seeding density. Note that K562 cells were non-adherent on the microwell array in the absence of a stencil. The data were pooled from three independent experiments. Error bars indicate SEM

The ability to efficiently pattern cells to defined locations is of significant value in single cell analysis. With this in mind, we next sought to investigate the frequency of single cell occupancy in microwells seeded through our stencil. The relationship between the seeding density and the percentage of sampled wells containing single cells (single cell occupancy) was found to exhibit different trends for each cell line. The K562 cell line, which requires the use of a stencil to achieve successful adhesion, exhibited very little correlation between seeding density and single cell occupancy, which remained at approximately 17% through all experimental densities (Fig. 5). Single cell occupancies of HME1 cells trended toward a loosely parabolic dependence on seeding density, with densities near 50 K cells/mL yielding the largest fraction of single-cell wells for both stencil seeded and control cells. Stencil-seeded and directly-seeded HME1 cells did not differ significantly in single cell occupancy (Mann-Whitney U test: *p* > 0.1) (Fig. 5). In contrast, single cell occupancies of MDA-MB-231 were positively impacted by stencil-seeding. MDA-MB-231 stencil-seeded substrates with seeding densities above 50 K cells/mL were found to be significantly higher (Mann-Whitney U test: *p* < 0.05) than directly-seeded controls, averaging at about 31% single cell occupancy. We observed that at high concentrations, these cells tend to clump together into loosely adherent aggregates. We therefore speculate that the stencil acts as a cellular sieve, favoring single cells or doublets, while inhibiting the passage of larger aggregates. The exhaustive distributions of empty, single, double, triple, and quadruple occupied wells under various seeding conditions was also investigated (Fig. 6). These data support the idea that the stencil shifts the balance of cell distribution in wells toward single cells at the expense of wells containing four cells in the MDA -MB-231 cell line.

## Discussion

Cell patterning has broad applications, particularly in the field of single cell analysis. Though many approaches have been described, limitations remain such as expense, difficulty of use, and cell stress. The stencil method presented here resolves these challenges. We have implemented this approach in conjunction with single cell metabolic analysis. In related work, our group has developed a platform for high throughput analysis of single cell oxygen consumption rate and extra cellular acidification [67]. In this approach a microwell array, as described here, is seeded with cells, then sealed with a lid coated with an oxygen and pH sensitive, fluorescent sensor film [68–71]. Each well is temporarily, hermetically sealed with the sensor lid, allowing measurement of metabolic output from individual cells or small groups of interacting cells. For this particular application, exclusion of cells from the microwell lips is critical for effective sealing of the array as well as reducing cellular responses to components released from damaged cells caught between the sealing surfaces. After random seeding through the stencil, we employ automated image analysis to determine the number of cells in each well and correlate the number of cells with metabolic readouts.

While higher single cell occupancy, approaching 100%, can be achieved by modern microfluidic methods [72], the use of a stencil has the benefit of not subjecting the cells to the high shear stress environments or impacts characteristic of high-throughput microfluidic devices. Biocompatibility of the stencil was demonstrated using a LIVE/DEAD assay, which indicated no increase in cell death. Cell viability was comparable to what has been previously reported for surface affinity patterning at 95% [50], micro-trap patterning at 94% [25], cell deposition patterning at 90% [19], and block-cell patterning at 95% [23] for various cell lines.

An additional advantage of our stencil approach is that it separates the cell patterning function from downstream analysis or additional experimental manipulations. For example, the design of our metabolic analysis platform [67], is optimized for sealing the microwells for efficient metabolite measurements. Engineering additional functionality for cell trapping such as a microfluidic component, would complicate both the device fabrication and experimental protocol, as well as likely compromise the analytic functionality. The stencil design also dramatically simplifies the cell loading procedure for the end user. Microfluidic-based cell patterning requires specialized equipment, expertise, and relatively demanding optimization. Use of the patterning stencil described here, requires only standard cell culture skills and no specialized or costly equipment.

Though we describe a specific application, this general approach, a removable stencil fabricated in situ on a functional substrate, is extensible to a wide range of applications and geometries. In recent years there have been remarkable advances in lab-on-chip and high throughput single cell analytic technologies [73]. Many of these platforms require precise localization of individual cells prior to cell lysis [74] or on-chip analyses such as qPCR [75, 76], proteomics [77], genome sequencing [78] or cell signaling [79]. Integrating stencil-assisted cell seeding into these emerging technologies has the potential to simplify device fabrication and dramatically reduce the complexity of experimental protocols. This method retains the ease of random cell seeding, with equivalent or increased single cell deposition, while dramatically reducing the occurrence of “off-target” cells.

## Conclusions

We demonstrate a laser-fabricated cell seeding stencil to be highly effective at patterning cells into features of microfabricated substrates. Stencils are readily adaptable to specific substrates or experimentally desirable geometries and multiple cell types. This approach does not expose cells to any physical stresses beyond those of standard cell culture. Since the cell stencil approach relies on a physical barrier instead of differential cell adhesion to achieve isolation, it does not select subpopulations of cells based on adhesion properties or expose cells to reactive substrates that may alter cellular physiology. Preparation of the stencil is simple and high throughput; stencils for 2980-microwell arrays can be prepared in less than an hour. Because the stencil is fabricated while affixed to the cell seeding substrate, there is no need for microscale stencil-substrate alignment, dramatically simplifying its usage. Since the specificity of cell localization is based on the design of the stencil and not the cell seeding technique, this approach is readily accessible to researchers in biological fields using standard cell culture techniques.

## References

[1] Dimov IK, Lu R, Lee EP, Seita J, Sahoo D, Park SM, Weissman IL, Lee LP. Discriminating cellular heterogeneity using microwell-based RNA cytometry. Nat Commun. 2014;5. doi:10.1038/ncomms4451. 10.1038/ncomms4451 PMC4075946 24667995

[2] Macosko EZ, Basu A, Satija R, Nemesh J, Shekhar K, Goldman M, Tirosh I, Bialas AR, Kamitaki N, Martersteck EM (2015). Highly parallel genome-wide expression profiling of individual cells using Nanoliter droplets. Cell.

[3] Streets AM, Zhang XN, Cao C, Pang YH, XL W, Xiong L, Yang L, YS F, Zhao L, Tang FC (2014). Microfluidic single-cell whole-transcriptome sequencing. Proc Natl Acad Sci U S A.

[4] White AK, Heyries KA, Doolin C, VanInsberghe M, Hansen CL (2013). High-throughput microfluidic single-cell digital polymerase chain reaction. Anal Chem.

[5] Guillotin B, Guillemot F (2011). Cell patterning technologies for organotypic tissue fabrication. Trends Biotechnol.

[6] Hribar KC, Meggs K, Liu J, Zhu W, Qu X, Chen SC. Three-dimensional direct cell patterning in collagen hydrogels with near-infrared femtosecond laser. Sci Rep. 2015;5 10.1038/srep17203 PMC4658636 26603915

[7] Jaganathan H, Gage J, Leonard F, Srinivasan S, Souza GR, Dave B, Godin B. Three-dimensional in vitro co-culture model of breast tumor using magnetic levitation. Sci Rep. 2014;4. doi:10.1038/srep06468. 10.1038/srep06468 PMC4180823 25270048

[8] Kellogg RA, Gomez-Sjoberg R, Leyrat AA, Tay S (2014). High-throughput microfluidic single-cell analysis pipeline for studies of signaling dynamics. Nat Protoc.

[9] Xu F, Celli J, Rizvi I, Moon S, Hasan T, Demirci U (2011). A three-dimensional in vitro ovarian cancer coculture model using a high-throughput cell patterning platform. Biotechnol J.

[10] Heath JR, Ribas A, Mischel PS (2016). Single-cell analysis tools for drug discovery and development. Nat Rev Drug Discov.

[11] Kang DK, Gong XQ, Cho S, Kim JY, Edel JB, Chang SI, Choo J, Demello AJ (2015). 3D droplet microfluidic Systems for High-Throughput Biological Experimentation. Anall Chem.

[12] Neuzil P, Giselbrecht S, Lange K, Huang TJ, Manz A (2012). Revisiting lab-on-a-chip technology for drug discovery. Nat Rev Drug Discov.

[13] Chen YC, Allen SG, Ingram PN, Buckanovich R, Merajver SD, Yoon E (2015). Single-cell migration Chip for Chemotaxis-based microfluidic selection of heterogeneous cell populations. Sci Rep.

[14] Hulkower KI, Perr M (2008). Quantifying cell migration and invasion. Genet Eng Biotechn N.

[15] Greaves M, Maley CC (2012). Clonal evolution in cancer. Nature.

[16] Lidstrom ME, Meldrum DR (2003). Life-on-a-chip. Nat Rev Microbiol.

[17] Marusyk A, Almendro V, Polyak K (2012). Intra-tumour heterogeneity: a looking glass for cancer?. Nat Rev Cancer.

[18] Pietras K, Ostman A (2010). Hallmarks of cancer: interactions with the tumor stroma. Exp Cell Res.

[19] Gross A, Schondube J, Niekrawitz S, Streule W, Riegger L, Zengerle R, Koltay P (2013). Single-cell printer: automated, on demand, and label free. Jala- J Lab Autom.

[20] Stumpf F, Schoendube J, Gross A, Rath C, Niekrawietz S, Koltay R, Roth G, Single-cell PCR (2015). Of genomic DNA enabled by automated single-cell printing for cell isolation. Biosens Bioelectron.

[21] Liberski AR, Delaney JT, Schubert US (2011). “eOne cell-one well”: a new approach to inkjet printing single cell microarrays. ACS Comb Sci.

[22] Martinez V, Forro C, Weydert S, Aebersold MJ, Dermutz H, Guillaume-Gentil O, Zambelli T, Voros J, Demko L (2016). Controlled single-cell deposition and patterning by highly flexible hollow cantilevers. Lab Chip.

[23] Zhang K, Chou CK, Xia XF, Hung MC, Qin LD (2014). Block-cell-printing for live single-cell printing. Proc Natl Acad Sci U S A.

[24] Wlodkowic D, Faley S, Zagnoni M, Wikswo JP, Cooper JM (2009). Microfluidic single-cell Array Cytometry for the analysis of tumor apoptosis. Anal Chem.

[25] Chung KH, Rivet CA, Kemp ML, Lu H (2011). Imaging single-cell signaling dynamics with a deterministic high-density single-cell trap Array. Anal Chem.

[26] Jin D, Deng B, Li JX, Cai W, Tu L, Chen J, Wu Q, Wang WH. A microfluidic device enabling high-efficiency single cell trapping. Biomicrofluidics. 2015;9(1) 10.1063/1.4905428 PMC4288539 25610513

[27] Barty A, Caleman C, Aquila A, Timneanu N, Lomb L, White TA, Andreasson J, Arnlund D, Bajt S, Barends TRM (2012). Self-terminating diffraction gates femtosecond X-ray nanocrystallography measurements. Nat Photonics.

[28] Kimmerling RJ, Szeto GL, Li JW, Genshaft AS, Kazer SW, Payer KR, Borrajo JD, Blainey PC, Irvine DJ, Shalek AK, et al. A microfluidic platform enabling single-cell RNA-seq of multigenerational lineages. Nat Commun. 2016;7 10.1038/ncomms10220 PMC4729820 26732280

[29] Ryley J, Pereira-Smith OM (2006). Microfluidics device for single cell gene expression analysis in Saccharomyces Cerevisiae. Yeast.

[30] PH W, Phillip JM, Khatau SB, Chen WC, Stirman J, Rosseel S, Tschudi K, Van Patten J, Wong M, Gupta S, et al. Evolution of cellular morpho-phenotypes in cancer metastasis. Sci Rep. 2015;5 10.1038/srep18437 PMC4682070 26675084

[31] Brouzes E, Medkova M, Savenelli N, Marran D, Twardowski M, Hutchison JB, Rothberg JM, Link DR, Perrimon N, Samuels ML (2009). Droplet microfluidic technology for single-cell high-throughput screening. Proc Natl Acad Sci U S A.

[32] Guo MT, Rotem A, Heyman JA, Weitz DA (2012). Droplet microfluidics for high-throughput biological assays. Lab Chip.

[33] Mazutis L, Gilbert J, Ung WL, Weitz DA, Griffiths AD, Heyman JA (2013). Single-cell analysis and sorting using droplet-based microfluidics. Nat Protoc.

[34] Wen N, Zhao Z, Fan BY, Chen DY, Men D, Wang JB, Chen J. Development of droplet microfluidics enabling high-throughput single-cell analysis. Molecules. 2016;21(7) 10.3390/molecules21070881 PMC6272933 27399651

[35] Varma S, Voldman J (2015). A cell-based sensor of fluid shear stress for microfluidics. Lab Chip.

[36] Ino K, Okochi M, Konishi N, Nakatochi M, Imai R, Shikida M, Ito A, Honda H (2008). Cell culture arrays using magnetic force-based cell patterning for dynamic single cell analysis. Lab Chip.

[37] Okochi M, Matsumura T, Honda H (2013). Magnetic force-based cell patterning for evaluation of the effect of stromal fibroblasts on invasive capacity in 3D cultures. Biosens Bioelectron.

[38] Sen M, Ino K, Ramon-Azcon J, Shiku H, Matsue T (2013). Cell pairing using a dielectrophoresis-based device with interdigitated array electrodes. Lab Chip.

[39] Valero A, Braschler T, Demierre N, Renaud P. A miniaturized continuous dielectrophoretic cell sorter and its applications. Biomicrofluidics. 2010;4(2) 10.1063/1.3430542 PMC2917879 20697593

[40] Wang HSM, Elango IS, Shetty RM, Teller W, Shabilla A (2013). Rotation of cells and cell clusters in culture Media for Optical Computed Tomography. 17th Int Conf Miniaturized. Syst Chem Life Sci.

[41] Kirkham GR, Britchford E, Upton T, Ware J, Gibson GM, Devaud Y, Ehrbar M, Padgett M, Allen S, Buttery LD, et al. Precision assembly of complex cellular microenvironments using holographic optical tweezers. Sci Rep. 2015;5. doi:10.1038/srep08577. 10.1038/srep08577 PMC4341216 25716032

[42] Kolb T, Albert S, Haug M, Whyte G (2014). Dynamically reconfigurable fibre optical spanner. Lab Chip.

[43] Mirsaidov U, Scrimgeour J, Timp W, Beck K, Mir M, Matsudaira P, Timp G (2008). Live cell lithography: using optical tweezers to create synthetic tissue. Lab Chip.

[44] Rasmussen MB, Oddershede LB, Siegumfeldt H (2008). Optical tweezers cause physiological damage to Escherichia Coli and Listeria bacteria. Appl Environ Microb.

[45] Collins DJ, Morahan B, Garcia-Bustos J, Doerig C, Plebanski M, Neild A. Two-dimensional single-cell patterning with one cell per well driven by surface acoustic waves. Nat Commun. 2015;6:8686. doi:10.1038/ncomms9686. 10.1038/ncomms9686 PMC4659840 26522429

[46] Gesellchen F, Bernassau AL, Dejardin T, Cumming DRS, Riehle MO (2014). Cell patterning with a heptagon acoustic tweezer - application in neurite guidance. Lab Chip.

[47] Ahmed D, Ozcelik A, Bojanala N, Nama N, Upadhyay A, Chen YC, Hanna-Rose W, Huang TJ. Rotational manipulation of single cells and organisms using acoustic waves. Nat Commun. 2016;7. doi:10.1038/ncomms11085. 10.1038/ncomms11085 PMC4814581 27004764

[48] Shi JJ, Ahmed D, Mao X, Lin SCS, Lawit A, Huang TJ (2009). Acoustic tweezers: patterning cells and microparticles using standing surface acoustic waves (SSAW). Lab Chip.

[49] Azioune A, Storch M, Bornens M, Thery M, Piel M (2009). Simple and rapid process for single cell micro-patterning. Lab Chip.

[50] Flaim CJ, Chien S, Bhatia SN (2005). An extracellular matrix microarray for probing cellular differentiation. Nat Methods.

[51] Lan S, Veiseh M, Zhang MQ (2005). Surface modification of silicon and gold-patterned silicon surfaces for improved biocompatibility and cell patterning selectivity. Biosens Bioelectron.

[52] Ren D, Xia YQ, Wang J, You Z (2013). Micropatterning of single cell arrays using the PEG-Silane and Biotin-(Strept) Avidin system with photolithography and chemical vapor deposition. Sensor Actuat B-Chem.

[53] Li GN, Yang G, Zhang PC, Li YY, Meng JX, Liu HL, Wang ST (2015). Rapid cell patterning induced by differential topography on silica Nanofractal substrates. Small.

[54] Li W, Tang QY, Jadhav AD, Narang A, Qian WX, Shi P, Pang SW. Large-scale topographical screen for investigation of physical neural-guidance cues. Sci Rep. 2015;5:8644. doi:10.1038/srep08644. 10.1038/srep08644 PMC4345323 25728549

[55] Kaji H, Camci-Unal G, Langer R, Khademhosseini A (2011). Engineering systems for the generation of patterned co-cultures for controlling cell-cell interactions. Bba-Gen Subjects.

[56] Mooney R, Haeger S, Lawal R, Mason M, Shrestha N, Laperle A, Bjugstad K, Mahoney M (2011). Control of neural cell composition in poly(ethylene glycol) Hydrogel culture with soluble factors. Tissue Eng Pt A.

[57] Jeanes A, Gottardi CJ, Yap AS (2008). Cadherins and cancer: how does cadherin dysfunction promote tumor progression?. Oncogene.

[58] Steinberg MS (2007). Differential adhesion in morphogenesis: a modern view. Curr Opin Genet Dev.

[59] Berrier AL, Yamada KM (2007). Cell-matrix adhesion. J Cell Physiol.

[60] Glenn HL, Wang ZH, Schwartz LM (2010). Acheron, a lupus antigen family member, regulates integrin expression, adhesion, and motility in differentiating myoblasts. Am J Physiol-Cell Physiol.

[61] Dai W, Li WB, Ren KN, Wu HK (2016). Convenient, reliable, bias-free dynamic patterning of multiple types of cells into precisely defined micropatterns for co-culture study. Chem Aust.

[62] Etzkorn JR, WC W, Tian ZY, Kim P, Jang SH, Meldrum DR, Jen AKY, Parviz BA. Using micro-patterned sensors and cell self-assembly for measuring the oxygen consumption rate of single cells. J Micromech Microeng. 2010;20(9)

[63] Javaherian S, O'Donnell KA, McGuigan AP, Fast A. Accessible methodology for micro-patterning cells on standard culture substrates using Parafilm (TM) inserts. PLoS One. 2011;6(6) 10.1371/journal.pone.0020909 PMC3110254 21687691

[64] Li W, Xu Z, Huang JZ, Lin XD, Luo RC, Chen CH, Shi P. NeuroArray: a universal Interface for patterning and interrogating neural circuitry with single cell resolution. Sci Rep. 2014;4 10.1038/srep04784 PMC3998032 24759264

[65] JB W, Zhang MY, Chen LQ, Yu V, Wong JTY, Zhang XX, Qin JH, Wen WJ (2011). Patterning cell using Si-stencil for high-throughput assay. RSC Adv.

[66] Lew V, Nguyen D, Khine M (2011). Shrink-induced single-cell plastic microwell Array. Jala-J Lab Autom.

[67] Kelbauskas L, Glenn H, Anderson C, Messner J, Lee KB, Song GQ, Houkal J, FY S, Zhang LQ, Tian YQ, et al. A platform for high-throughput bioenergy production phenotype characterization in single cells. Sci Rep. 2017;7. doi:10.1038/srep45399. 10.1038/srep45399 PMC5368665 28349963

[68] HG L, Jin YG, Tian YQ, Zhang WW, Holl MR, Meldrum DR (2011). New ratiometric optical oxygen and pH dual sensors with three emission colors for measuring photosynthetic activity in cyanobacteria. J Mater Chem.

[69] Tian YQ, Shumway BR, Gao WM, Youngbull C, Holl MR, Johnson RH, Meldrum DR (2010). Influence of matrices on oxygen sensing of three sensing films with chemically conjugated platinum porphyrin probes and preliminary application for monitoring of oxygen consumption of Escherichia Coli (E coli). Sensor Actuat B-Chem.

[70] Tian YQ, Shumway BR, Youngbull AC, Li YZ, Jen AKY, Johnson RH, Meldrum DR (2010). Dually fluorescent sensing of pH and dissolved oxygen using a membrane made from polymerizable sensing monomers. Sensor Actuat B-Chem.

[71] Tian YQ, FY S, Weber W, Nandakumar V, Shumway BR, Jin YG, Zhou XF, Holl MR, Johnson RH, Meldrum DR (2010). A series of naphthalimide derivatives as intra and extracellular pH sensors. Biomaterials.

[72] Frimat JP, Becker M, Chiang YY, Marggraf U, Janasek D, Hengstler JG, Franzke J, West J (2011). A microfluidic array with cellular valving for single cell co-culture. Lab Chip.

[73] Conde JP, Madaboosi N, Soares RRG, Fernandes JTS, Novo P, Moulas G, Chu V (2016). Lab-on-chip systems for integrated bioanalyses. Biosensor Technologies for Detection of biomolecules. Edited by Estrela P, vol. 60.

[74] Nan L, Jiang ZD, Wei XY (2014). Emerging microfluidic devices for cell lysis: a review. Lab Chip.

[75] Dong H, Sun H, Zheng JP (2016). A microchip for integrated single-cell genotoxicity assay. Talanta.

[76] Zhang R, Gong HQ, Zeng XD, Lou CP, Sze C, Microfluidic Liquid A (2013). Phase nucleic acid purification Chip to selectively isolate DNA or RNA from low copy/single bacterial cells in minute sample volume followed by direct on-Chip quantitative PCR assay. Anal Chem.

[77] Lee WC, Rigante S, Pisano AP, Kuypers FA (2010). Large-scale arrays of picolitre chambers for single-cell analysis of large cell populations. Lab Chip.

[78] Gole J, Gore A, Richards A, Chiu YJ, Fung HL, Bushman D, Chiang HI, Chun J, Lo YH, Zhang K (2013). Massively parallel polymerase cloning and genome sequencing of single cells using nanoliter microwells. Nat Biotechnol.

[79] McWhorter FY, Smith TD, Luu TU, Rahim MK, Haun JB, Liu WF (2016). Macrophage secretion heterogeneity in engineered microenvironments revealed using a microwell platform. Integr Biol.

